# Lipoprotein Lipase Activity Does Not Differ in the Serum Environment of Vegans and Omnivores

**DOI:** 10.3390/nu15122755

**Published:** 2023-06-15

**Authors:** Natjan-Naatan Seeba, Robert Risti, Aivar Lõokene

**Affiliations:** Department of Chemistry and Biotechnology, Tallinn University of Technology, 12618 Tallinn, Estonia; naseeb@taltech.ee (N.-N.S.); robert.risti@taltech.ee (R.R.)

**Keywords:** vegan, omnivore, lipoprotein lipase, isothermal titration calorimetry

## Abstract

Although vegan diets have been reported to be associated with a reduced risk of cardiovascular disease, it was not known whether this might be partly due to vegan diets’ effects on plasma triglyceride metabolism. This study aimed to investigate if there are differences in the activity of lipoprotein lipase (LPL), an enzyme that functions at the vascular endothelium and is responsible for triglyceride breakdown, in sera obtained from vegans and omnivores. LPL activity was assessed using isothermal titration calorimetry, which allows measurements in undiluted serum samples, mimicking physiological conditions. Fasted sera from 31 healthy participants (12F 2M vegans, 11F 6M omnivores) were analyzed. The results indicated no significant differences in average LPL activity between the vegan and omnivore groups. Interestingly, despite similar triglyceride levels, there were considerable variations in LPL activity and total very-low-density lipoprotein triglyceride breakdowns between individuals within both groups. Biomarker analysis showed that vegans had lower total cholesterol and LDL-C levels compared to omnivores. These findings suggest that the lipid-related benefits of a vegan diet, in terms of atherogenic risk, may primarily stem from cholesterol reduction rather than affecting serum as a medium for LPL-mediated triglyceride breakdown. In healthy individuals, lipid-related changes in serum composition in response to a vegan diet are likely overshadowed by genetic or other lifestyle factors.

## 1. Introduction

Vegan diets are defined as diets that exclude all animal foods. Many studies show that a vegan diet reduces the risk of cardiovascular disease (CVD). This health benefit is generally thought to be related to the fact that vegans tend to have lower levels of plasma cholesterol, LDL-C and inflammatory biomarkers compared to people on omnivorous diets [1]. Less research has been carried out on how a vegan diet affects blood triglycerides (TG), elevated levels of which are also associated with CVD [2,3,4]. The results of prior studies suggest that fasting TG levels depend on the macronutrient composition of the diet: TGs are increased in low fat vegan diets and lowered in low carbohydrate vegan diets [1]. However, the noted effects of a vegan diet on the mechanisms involved in TG metabolism have been inconsistent [1,4].

One crucial factor in regulating TG levels in blood is the hydrolytic degradation of TGs in triglyceride-rich lipoproteins (TRL) by the catalytic action of endothelial-bound lipoprotein lipase (LPL). LPL also facilitates the cellular uptake of lipoprotein particles and cholesteryl esters [5]. LPL activity is regulated post-translationally by several proteins, such as apolipoproteins (Apo) and the angiopoietin-like proteins 4, -8 and -3 (ANGPTL4, -8 and -3). ApoC-II is a co-factor that acts as an activator for LPL, while ApoC-I and ApoC-III are known inhibitors of this enzyme.

Previous studies suggest a possible connection between LPL activity and nutrition [6,7,8,9]. Firstly, changes in diet can affect LPL activity through alternations in plasma concentrations of ANGPTL 3, -4 and 8 [10,11,12]. It has been proposed that the combined actions of these proteins regulate LPL activity in a tissue-specific manner and according to nutritional status. ANGPTL3, expressed in the liver, mildly inhibits LPL, and maintains consistent expression during fasting and eating. ANGPTL4, a potent LPL inhibitor, is predominantly present in fat, liver, intestine and heart tissues. During fasting, ANGPTL4 is highly expressed in adipose tissue, and the comparatively lower inhibitory impact of circulating ANGPTL3 ensures LPL remains active in skeletal muscle but inactive in adipose tissue. This promotes the breakdown of TRLs in skeletal muscles, providing fatty acids for energy production. ANGPTL8, primarily expressed in the liver and fat, can form complexes with ANGPTL4 and ANGPTL3, and its independent LPL inhibitory activity is minimal. ANGPTL8 expression is low during fasting but, stimulated by insulin, significantly increases while eating. The ANGPTL3/8 complex has a stronger inhibitory effect than free ANGPTL3, while the ANGPTL4/8 complex has a weaker effect than free ANGPTL4. This mechanism aims to direct TRL breakdown to adipose tissue after a meal, facilitating the conversion of released fatty acids into TGs [13,14,15]. Another dietary factor that can affect LPL lipolysis is the composition of TRLs. LPL has been shown to favor TRLs, which are predominantly composed of unsaturated fatty acids [16,17,18]. Studies focusing on the serum fatty acid profile of vegans and omnivores show that vegans have higher levels of (*n*-6) PUFA, (*n*-9) PUFA, and α-linolenic acid (ALA) and lower levels of *trans*-fatty acids, saturated fatty acids (SFA), docosahexaenoic acid (DHA) and eicosapentaenoic acid (EPA) [19]. The supplementation of omega 3-fatty acids, e. g. EPA and DHA, increases the marginalization values of TRL particles, suggesting that these fatty acids may increase the amount of endothelial-bound LPL or its affinity for TRLs [17]. Although these observations suggest a link between the regulation of LPL activity and a vegan diet, this connection has received little study. Vinagre et al. compared TRL metabolism and lipid transfer to HDL particles in vegans and omnivores using a synthetic radio-labeled substrate. No differences in post-heparin LPL activity and plasma lipolysis kinetics were found, but a higher clearance of atherogenic remnants was observed in vegans [20].

The efficacy of endothelial lipolysis by LPL depends on two factors: (a) the amount of active LPL on the endothelium and (b) the total effect of various extracellular regulators on LPL activity. The activity and amount of LPL bound to the endothelium can be determined from post-heparin plasma. The intravenous administration of heparin releases LPL from its endothelial binding sites, resulting in the appearance of LPL in blood circulation. Vinagre et al. [20] have shown that a vegan diet does not affect the post-heparin activity of LPL. However, it was unknown how a vegan diet changes the composition of the blood plasma environment as a medium of lipolysis.

In the present study, we investigated if the vegan diet has an effect on serum components that may influence LPL activity, when compared to omnivores. We measured LPL activity using a previously developed method that is based on isothermal titration calorimetry (ITC method). Our method makes it possible to perform measurements directly in undiluted human blood plasma or serum without the need for synthetic radioactive/fluorogenic substrates. This avoids the isolation of TRLs, which can potentially damage their structure and apolipoprotein composition. The ITC method measures heat produced by LPL during TRL hydrolysis, which is proportional to the amount of fatty acids released [21]. Thus, it is an appropriate method for studying the blood serum or plasma of subjects as an environment for LPL-mediated TRL hydrolysis and for comparing whether the blood sera of vegans and omnivores affect LPL differently. We observed no significant difference in mean LPL activity measured in the sera of omnivores and vegans. At the same time, measurements revealed participants in both groups had the same level of serum TGs, where the measured LPL activity differed by several fold. We conclude that factors other than diet play a more important role in the regulation of LPL activity. The results of this study also suggest that the lipid-related anti-atherogenicity of the vegan diet is limited to cholesterol lowering.

## 2. Materials and Methods

### 2.1. Participants

This study was approved by the Human Research Ethics Committee of the Estonian Institute for Health Development (Decision nr 1044, protocol code 36 date of approval 16.02.2022). Fourteen healthy normal-weight vegans and seventeen healthy normal-weight omnivores gave their informed consent to be enrolled in the study and were assigned codes that systematically maintained anonymity for all clinical data. Subjects in both groups fell within similar parameters. The average age of the vegan subjects was 29 ± 6 year and the average age of the omnivore subjects 27 ± 5 year. The vegan group consisted of 12 female and 2 male subjects, while the omnivore group consisted of 6 male and 11 female subjects. All participants were instructed to avoid alcohol consumption during the seven-day period. Participants in the vegan group were strict vegetarians and did not consume any fish, eggs, or dairy products. Subjects in the control group were not following any specific restrictive diet prior to the study, consumed both animal-derived food and plant-based food, and were instructed not to change their habitual diets. Exclusion criteria for participants were the following: restrictive diets (n/a vegan diets), usage of prescription medications, pre-existing health conditions that could influence metabolism, i.e., metabolic syndrome, being overweight, and intense physical activity (physical activity level PAL < 1.99). Participants were asked to fill out a seven-day food diary to determine their diet compositions and were instructed to donate blood after a 12 h fast on the eighth day. The blood sera of participants were then used to measure LPL activity and several blood biomarkers. This study was carried out in accordance with the Declaration of Helsinki.

### 2.2. Reagents

Blood was drawn from vegan and omnivore participants after fasting, and the serum was separated by SYNLAB Estonia Ltd. (Tallinn, Estonia) TG, LDL-C, HDL-C, total cholesterol (TC), apolipoprotein A-I (ApoA-I) and apolipoprotein B (ApoB) levels were also determined by SYNLAB Estonia Ltd. using the following methods: ApoB and ApoA-I were measured using nephelometry (Atellica Neph 630); TG, LDL-C, HDL-C and TC were measured using photometric kits Alinity c (Abbott (Abbott Park, IL, USA)). Serum samples were frozen and stored at −80 °C. Samples were thawed immediately before determining LPL activity. Reference plasma samples were purchased from Tallinn Blood Centrum and stored at −80 °C. LPL was purified from bovine milk as described previously [22] and dialyzed against 1 M NaCl and 20 mM bis-Tris, pH 7.4. Bovine serum albumin (BSA) (#A7906) was purchased from Sigma Aldrich (St. Louis, MO, USA)and heparin (#411212500) was purchased from Acros Organics. The Superose 6 10/300 GL increase 24 mL column used for lipoprotein separation was purchased from Sigma Aldrich.

### 2.3. Isothermal-Titration Calorimetry (ITC) Measurements for LPL Activity

**Figure 1:**
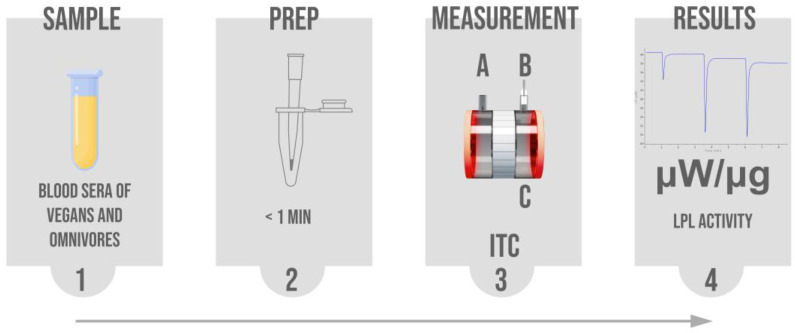
General step-by-step workflow of the applied ITC method for LPL activity measurements. The received blood sera of vegan and omnivore participants (1) were prepared (2) for insertion in the isothermal titration calorimeter measurement cell (C). (3) During the measurements, the burette (B) contained an LPL enzyme mixture, while the ITC reference cell (A) contained MiliQ water. While constantly stirring, a series of LPL injections were made into the measurement cell (C) containing the blood serum of the subject. Lipolysis was monitored and the results were obtained as heat released over time. The results were then calculated to changes in heat rate per µg of protein, which was proportional to the amount of fatty acids release per µg of protein (4).

Experiments were performed on a MicroCal PEAQ-ITC (Malvern) as previously described [23]. Measurements of LPL activity in the participants’ blood sera were made against a reference measurement of human blood plasma with a TG concentration of 1.25 mM. Reference measurements were made randomly in between standard measurements to assess and eliminate day-to-day variations in LPL activity. In a standard measurement, the calorimetric measurement cell was filled with the participants blood serum and 7 µL of 20 mM Tris pH 7.4 buffer mixture. The syringe contained 200 nM LPL in 150 mM NaCl, 20 mM HEPES, pH 7.4 buffer with 50 mg/mL BSA and 10 IU/mL heparin. Albumin and heparin were added to ensure the catalytic stability of the LPL [24]. The calorimetric reference cell contained MilliQ water. All conditions were the same in the reference measurements as in the standard measurements, except the blood serum of the participant was replaced with reference blood plasma. The stirring speed in the measurement cell was 1000 rpm in all experiments. A series of four injections of the LPL mixture were made into the measurement cell, where the first injection was 0.4 μL and the three subsequent injections were 1 μL. The changes in heat released were recorded for 150 s following each injection. The first injection was always excluded from data analysis. After each measurement, the syringe and sample cell were washed with 10% Decon 90 and rinsed with MilliQ water. Methanol was used to additionally rinse the syringe before every experiment. The LPL activities of the measurements in participants’ sera were normalized against the LPL activities in the reference measurements. The measured LPL activity is expressed as released heat rate in µW per µg of protein, which is proportional to fatty acids released per µg of protein [23]. The workflow of the method is graphically illustrated in Figure 1.

### 2.4. Evaluating the Efficacy of Long-Term Lipolysis Using Size Exclusion Chromatography (SEC) after Treatment with LPL

Blood sera from participants were incubated in 1.5 mL tubes at room temperature in an Eppendorf ThermoMixer, shaking at 300 rpm overnight with a 20 mM tris pH 7.4 buffer, either with 40 nM LPL or MilliQ water (blank). The incubation parameters and LPL concentration were selected to ensure total hydrolysis of the TG substrate that was available to the enzyme. We had previously shown via ITC that an LPL concentration of 10 nM was enough to guarantee total substrate hydrolysis after two hours of initial enzyme injection [21]. Lipoprotein fractionation before and after incubation with LPL was performed on an Äkta purifier 10 system using a Superose 6 10/300 GL column equilibrated with two column volumes (CV) of 20 mM Tris, 0.15 M NaCl, 1 mM EDTA pH 7.4 buffer. The flow rate was 0.5 mL/min and the volume of elution buffer used was 1.3 CV. Human serum albumin (HSA) and lipoprotein fractions obtained using density gradient ultracentrifugation, as described previously [25], were used as reference standards. The before and after incubation chromatograms of participants were normalized to the HSA peak for each individual and the percentage changes in the VLDL peaks of the samples were compared.

### 2.5. Data Analysis

An XLSTAT principal component analysis (PCA) was used to compare the measured blood parameters, average daily consumption of fatty acids, rate of VLDL hydrolysis and LPL activities. A Pearson’s correlation coefficient was calculated to determine the correlation between variables. Variables were analyzed using a Student’s *t* test, and analyses that yielded Ps ≤ 0.05 were considered to be statistically significant. The results are presented as means ± SEM. The National Institute for Health Development’s food composition and the USDA (U.S Department of Agriculture) food composition databases were used to estimate the average daily fatty acid consumption of participants based on the food diary entries. The changes in the area of VLDL peaks in chromatograms were calculated using a custom written code available through Google colab (Appendix A).

## 3. Results

### 3.1. Ad Libitum Energy Intake and Macronutrient Composition of Both Diets

**Figure 2:**
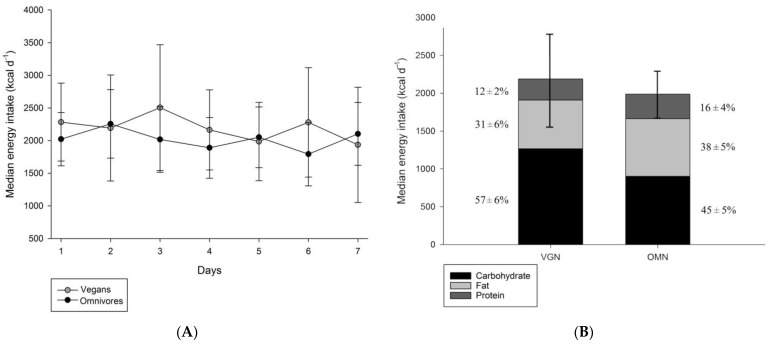
Ad libitum energy intake and median macronutrient composition of both groups: (**A**) average energy intake of both groups during the seven-day food diary recording period. No significant difference in the median daily energy intake was noted between the vegan (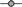) and omnivore (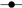) participants; (**B**) Median macronutrient composition of both diets illustrating the carbohydrate rich diet of the vegan group > 45% of energy from carbohydrates and the fat-rich diet of the omnivore group > 35% of energy from fats. On average, vegan participants consumed more carbohydrates and less protein and fat than their omnivore counterparts.

During the seven-day period of food diary recording, there was no statistically significant difference in ad libitum energy intake between the vegan and omnivorous groups; the mean energy intakes were 2189 ± 624 kcal d^−1^ in the vegan group and 1989 ± 318 kcal d^−1^ in the omnivorous group (*p* = 0.41) (Figure 2A). However, the diets differed in macronutrient composition: the vegan group consumed an average of 31 ± 6% fat, 57 ± 6% carbohydrate, and 12 ± 2% protein, while the omnivorous group consumed an average of 38 ± 5% fat, 45 ± 5% carbohydrate, and 16 ± 4% protein. (Figure 2B). Thus, on average, the omnivorous group consumed 21% more fat (*p* < 0.05), 19% more protein (*p* < 0.05), and 21% fewer carbohydrates (*p* < 0.05) than the vegan group. Considering the macronutrient intake, the vegan group can be described as following a high-carbohydrate diet (more than 45% calories from carbohydrates) and the omnivore group can be described as following a high-fat diet (more than 35% calories from fats) [26,27]. The main sources of dietary fats for the vegan group consisted of a variety of nuts and seeds, olive oil, and avocados, while the main sources of dietary fats for the omnivores consisted of dairy products, red meat, poultry, and fish (Figure S1).

### 3.2. Comparison of Lipoprotein Parameters and LPL Activity

No significant difference in TG, HDL-C, ApoB or ApoA-I levels was detected between the vegan and omnivorous groups. The mean measured LPL activity was also similar: 56.53 ± 17.97 µW/µg for vegans and 57.26 ± 18.03 µW/µg for omnivores. However, there was a significant difference in LDL-C and TC levels: vegan participants had an average of 23% lower LDL-C and 14% lower TC levels compared to their omnivore counterparts (Table 1). The average TC value was 4.12 ± 0.82 mmol/L in vegans and 4.81 ± 0.85 mmol/L in omnivores.

**Figure 3:**
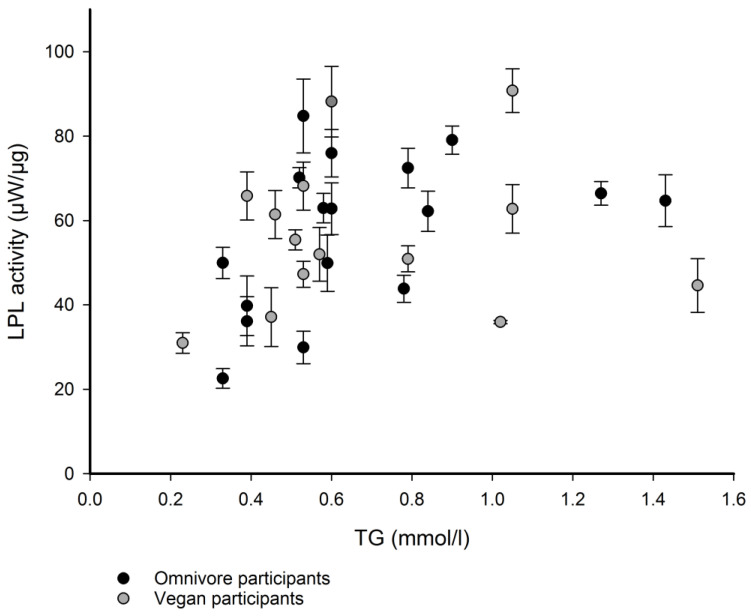
Scatter plot of measured exogenous LPL activities against TG concentration, N = 31. Data for LPL activity in the blood serum of each participant are presented as the mean of three independent measurements. Data for vegan sera are shown in gray and data for omnivorous participants are in black. LPL activity is expressed as heat rate per LPL mass (µW/µg), which is proportional to fatty acids released per µg of LPL.

Although there was no significant difference in mean LPL activities between the groups (Figure 3), both groups had participants with similar TG levels but significantly different LPL activity (Table 2), regardless of diet. The largest, a 2.8-fold, difference in LPL activity, 84.77 ± 8.76 µW/µg and 29.91 ± 3.85 µW/µg was detected in the blood sera of two omnivores, while a 2.5-fold difference (90.77 ± 5.17 µW/µg vs. 35.93 ± 0.39 µW/µg) in LPL activities was measured in two vegans. The largest difference in LPL activity measured in the sera of participants with similar TG levels but different diets was 1.8-fold (65.83 ± 5.70 µW/µg vs. 36.10 ± 5.82 µW/µg), where recorded LPL activity was higher in the vegan participant. These results suggest that factors other than diet are more important in regulating exogenous LPL activity in serum.

**Figure 4:**
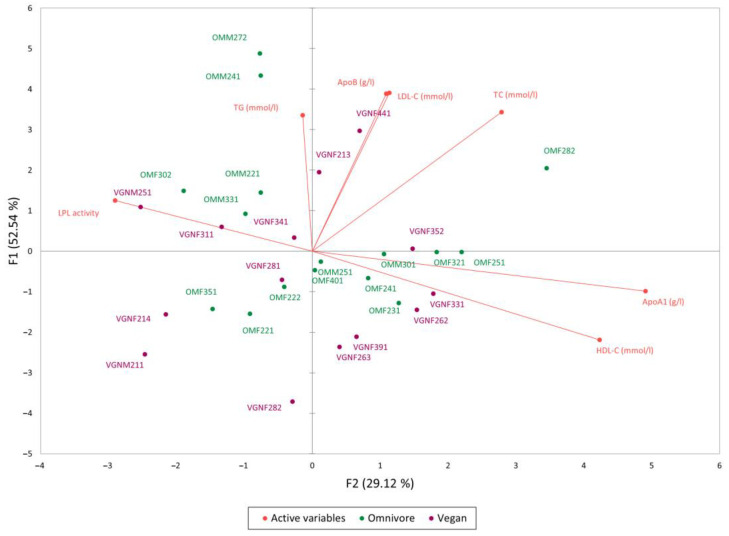
PCA loading plots of LPL activity and lipoprotein-associated blood parameters. In total, 81.66% of the variance is explained through two principal components: F1 (52.54%) and F2 (29.12%). Variables are depicted as vectors with the angle between vectors representing correlation. An acute angle between variables represents a positive correlation, while a right angle and obtuse angle represent no correlation and negative correlation, respectively. Omnivore participants are indicated in green and vegans in violet. Homogeneity in participants showed no difference in parameters except for TC and LDL-C, where omnivore participants were shifted towards higher values of the mentioned active variables.

**Figure 5:**
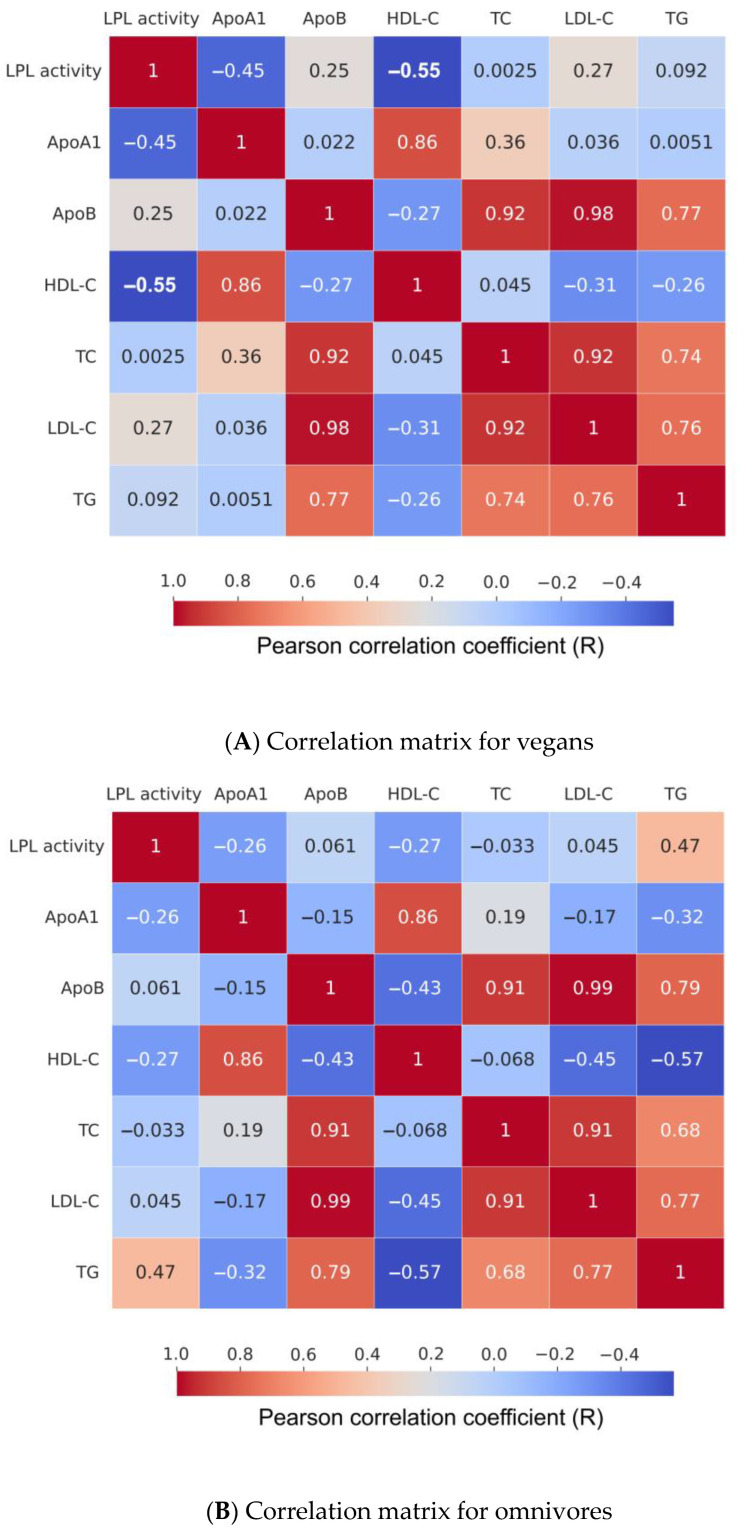
Correlation matrices of LPL activity and lipoprotein-associated blood parameters of both groups. (**A**) Pearson correlation matrix of vegan participants; a statistically significant negative correlation, r = −0.55 (*p* = 0.039), was found between measured LPL activity and HDL-C concentration (highlighted in bold). (**B**) Pearson correlation matrix of omnivore participants; unlike in vegans, a positive but statistically insignificant correlation of r = 0.47 between measured LPL activity and TG levels (*p* = 0.06) was noted in omnivore participants.

A principal component analysis (PCA) was used to better understand whether there was a relationship between the measured serum parameters and LPL activity (Figure 4). The PCA showed a negative correlation between HDL-C (r = −0.548) *p* = 0.039 concentration and LPL activity measured in the vegan group. In omnivores, a slight but statistically insignificant positive correlation of r = 0.466 was found between TG concentration and LPL activity. No correlation was found between TC and measured LPL activity in either group. A slight and statistically insignificant negative correlation with LPL activity was also observed for ApoA-I concentrations in both groups, with r = −0.258 for omnivores and r = −0.452 for vegans. No correlation was observed between LPL activity, ApoB and LDL-C levels in omnivores, whereas a slight statistically insignificant positive correlation was found in ApoB (r = 0.249) and LDL-C (r = 0.269) levels in vegans (Figure 5).

### 3.3. Fatty Acid Saturation and Measured LPL Activity

**Figure 6:**
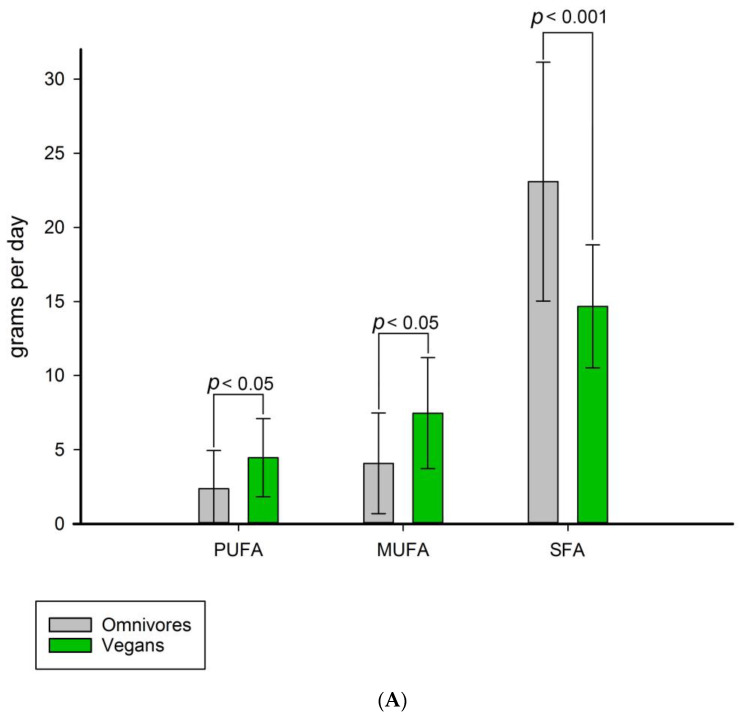

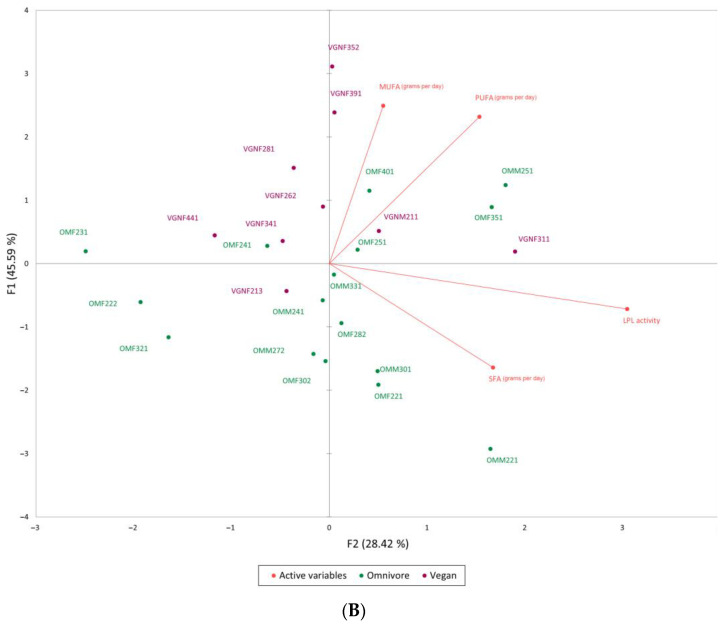
Average consumption of fatty acids and a PCA plot of LPL activity related to daily fatty acid intake. (**A**) SFA consumption was highest in both groups followed by PUFA and MUFA consumption. Vegans consumed more PUFA and MUFA than their omnivore counterparts; (**B**) The PCA loading plot shows no statistically significant correlation between daily fatty acid consumption and LPL activity (vegans are indicated in violet and omnivores in green).

The average daily fatty acid consumption was estimated based on data from participants’ food diaries and food composition databases. The daily saturated fat (SFA) intake was highest in both groups, with omnivores consuming 23.1 g per day and vegans 14.7 g per day. On average, vegan participants consumed more polyunsaturated fat (PUFA) and monounsaturated fat (MUFA) per day than omnivores: the differences were 4.5 ± 2.6 g per day for PUFA and 7.5 ± 3.7 g per day for MUFA. PUFA and MUFA consumption in omnivores was lower: 2.3 ± 2.5 g per day for PUFA and 4.1 ± 3.4 g per day for MUFA, respectively (Figure 6A). The PCA showed no statistically significant correlations between fatty acid consumption and measured LPL activity in either group. The corresponding Pearson correlation coefficients were as follows: SFA intake r = 0.594 for vegans (*p* = 0.11) and r = 0.268 for omnivores (*p* = 0.3); PUFA intake r = 0.111 for vegans (*p* = 0.7) and r = 0.2 for omnivores (*p* = 0.4). No correlation was found between MUFA consumption and the measured LPL activity (Figure 6B).

### 3.4. Evaluation of Long-Term Lipolysis Efficacy Using SEC

**Figure 7:**
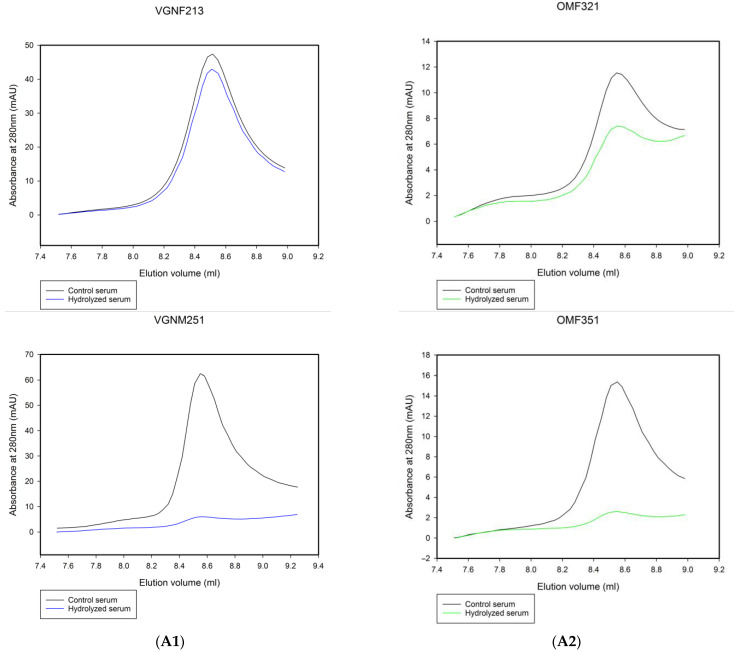

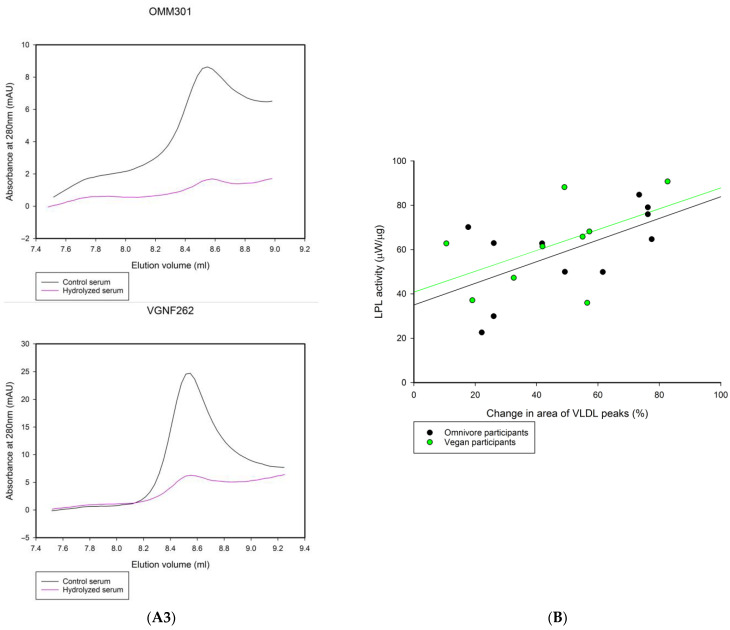
SEC analysis of long-term lipolysis. (**A1**–**A3**) Comparison of VLDL fractions for pairs of serum samples with similar TG levels before and after long-term lipolysis with LPL. The effects of treatment with LPL on the VLDL peaks for pairs with similar serum TG levels were as follows: (**A1**)—two vegans, VGNF213 and VGNM251, serum TG levels—1.05 mmol/L; (**A2**)—two omnivores, OMF321 and OMF351, serum TG levels—0.53 mmolL; (**A3**)—a vegan VGNF262 and an omnivore OMM301, serum TG levels of 0.52 mmol/L and 0.53 mmol/L, respectively. (**B**) Scatter plot showing the relationship between measured LPL activity and the percentage change in the area of VLDL peaks. Vegans are indicated in green and omnivores in black.

In the ITC measurements, we determined the initial rates of lipolysis to compare the serum of omnivores and vegans. However, the remodeling of lipoproteins and the clearance of circulating TGs in vivo can also depend on how much of the TG content in lipoproteins is accessible to LPL. The entry of large TRLs to the liver is limited [28]. The aim of the following experiments was to evaluate how much VLDL triglycerides could be hydrolyzed when the serum samples were incubated for a longer time with a higher amount of LPL. For this purpose, we fractionated lipoproteins before and after incubation with exogenous LPL using SEC. The percentage changes in VLDL peak areas were calculated and used as parameters for the evaluation of lipolysis efficacy. We observed a large variation in the percentage change in VLDL peak areas in both groups, but no significant difference between the two groups was found. The mean changes in VLDL peak areas were as follows: 45 ± 22% for vegans and 50 ± 24% for omnivores. Similarly to the ITC measurements, there were individuals in both groups with the same TG levels but different percentage changes in the areas of VLDL peaks. The largest difference in percentage change in VLDL among participants with the same TG level was between a 25-year-old male vegan (82.7% change) and a 21-year-old female vegan (10.6% change), with a difference of 72.1% (Figure 7A1). For omnivores with the same TG levels, the biggest difference in VLDL percentage change was between a 32-year-old female (26%) and a 35-year-old female (73.5%), with a difference of 47.5% (Figure 7A2). In the case of differing diets, the biggest difference in VLDL percentage change was 39.5% between a 30-year-old male omnivore (17.7%) and a 26-year-old female vegan (57.2%) (Figure 7A3). The correlation between initial LPL activity measured by ITC and lipolysis efficiency determined by SEC is presented in Figure 7B. The Pearson correlation coefficient for this relationship was r = 0.601 for omnivores and r = 0.525 (*p* > 0.05) for vegans, indicating that diet does not influence this dependence. These r values can be considered to show a moderate correlation between initial lipolysis rate and lipolysis efficiency [29].

## 4. Discussion

In the present study, we investigated whether a vegan diet and an omnivorous diet differentially affect the extracellular regulatory system of LPL. For this purpose, the activity of exogenous LPL in the blood serum of vegans and omnivores was compared using the ITC method developed by the laboratory of the authors of this study. The comparison of long-term lipolysis efficiency was evaluated after long-term serum incubation with LPL using SEC. In both cases, measurements were made in undiluted serum samples, i.e., under conditions that closely mimic the in vivo lipolysis environment. We observed no significant differences between the groups in serum TG levels, mean LPL activity, or long-term lipolysis efficiency. We conclude that the lipolysis of lipoproteins by LPL occurs equally well in the sera of omnivores and vegans. In other words, vegan diets do not affect the extracellular regulatory system of LPL differently than in omnivores. If we also take into account that the vegan diet does not affect the post-heparin activity of LPL, as has been demonstrated by Vinagre et al. [20], it can be argued that the vegan diet does not significantly affect the metabolism of TGs in blood. However, it should be noted that this conclusion regarding the regulatory system of LPL applies only to the subjects of this study: healthy individuals 21–44 years of age. Future studies are needed to elucidate whether a vegan diet affects TG lipolysis differently than in omnivores with other groups of people.

Although no differences were found in mean LPL activity between the vegan and omnivore groups, there were individuals in both groups with similar TG levels but largely different LPL activities. Additionally, an SEC analysis revealed participants with similar TG levels but vastly different changes in VLDL peaks after long-term lipolysis. The similar variations in both groups suggest that factors other than differences in diet play a more important role in the regulation of LPL activity. Our results can be compared to Vinagre et al.’s study in which the in vivo lipolysis of a synthetic substrate in the form of radio-labeled glycerol tri[3H]oleate was investigated. Although our results are largely in accordance with Vinagre et al.’s results, it is important to point out the necessity of our study. This is primarily related to the questionability of using artificial synthetic substrate emulsions. As previously shown, exchangeable apolipoproteins can attach to synthetic emulsion particles [30,31,32,33], but non-exchangeable apolipoproteins, such as ApoB48 in chylomicrons or ApoB100 in VLDL particles, remain bound to lipoproteins [34]. Therefore, synthetic emulsion particles are not identical to lipoprotein particles, and their fate in circulation can be different. In contrast to native lipoproteins, LPL has also been observed to have some basal ApoC-II independent activity against synthetic emulsions [35]. Thus, the use of synthetic substrates affects the regulatory system and the activity of LPL and lipid homeostasis [36]. In contrast to Vinagre et al.’s study, where a synthetic substrate was used, our ITC assay for LPL activity allows for the use of native unmodified substrates in the form of TRLs.

Higher LPL activities were measured in participants whose daily fat consumption consisted mainly of saturated fats. However, several in vitro studies [7,17] have shown that LPL prefers unsaturated fatty acids in TG composition. Our study was conducted with fasted blood sera, where the dominating substrates for LPL are VLDL particles. While VLDL composition and size can be affected by a difference in fat consumption [37], only about 20% of dietary fats are incorporated into VLDL after eating [38]. This percentage could be too low to impact LPL activity, or the effect could be overshadowed by other LPL regulators. Although vegans, on average, consumed more PUFA than omnivores, the n3-fatty acid content of daily PUFA consumed by vegans may be too low to impact LPL activity. It has been previously shown that in the treatment of hypertriglyceridemia the efficacious dosage of omega 3-fatty acids is at least 3 g per day [39]. Furthermore, the body’s ability to convert ALA to EPA and DHA is limited. Only about 5% of ALA is converted to EPA, while less than 0.5% is converted to DHA [40]. This, coupled with the fact that vegan participants exclude fish from their diet, could explain why the measured exogenous LPL activities did not differ significantly even though the vegan group consumed more PUFAs. On the other hand, Lopez-Soldado et al. found that TG secretion in VLDL particles can be increased by delivering saturated or n6- polyunsaturated fatty acids containing fats to hepatocytes via chylomicron remnant-like particles [37]. Considering that lipoprotein size is a main determining factor in the rate of hydrolysis for LPL [16], the possible changes in VLDL size due to the increase in TG secretion could potentially explain why higher LPL activities were measured in participants who, on average, consumed more saturated and n6-polyunsaturated fats in both groups. PCA showed that lower LPL activities were measured in participants with higher HDL-C and ApoA-I concentrations. These results are similar to the results of Kovrov and colleagues, who found an inverse correlation between HDL size, HDL-C, ApoA-I concentrations and measured LPL activity [16]. The exact reason for this is still unclear. The inverse correlation between measured LPL activity and ApoA-I concentration is most likely due to ApoA-I being a component of HDL [41]. Despite the fact that no significant difference between the average LPL activities measured in vegan and omnivore blood sera was found, it has been shown that serum ANGPTL protein levels can be influenced by dietary changes [11]. Kovrov et al. found no correlation between serum ANGPTL levels and measured LPL activity, which could indicate that the ANGPTL concentrations in fasted blood serum are too low to influence LPL activity or ANGPTL’s regulatory effects are in the corresponding tissues as opposed to directly in the blood serum [16,42].

As in several previous studies [1,20,43], we found that vegans had, on average, lower LDL-C and TC levels than omnivores, supporting the view that vegan diets are less atherogenic than those of omnivores. We were not able to identify an association between LDL-C levels and LPL activity. Thus, the results of this study suggest that the possible lipid-related anti-atherogenicity of the vegan diet is limited to the cholesterol-lowering effect. The main limitations of this study were the limited sample size, the unavailability of a few food diaries from vegans, and the uneven received volume of blood sera for some participants, which reduced the data available to analyze potential connections between aspects contained in food diaries, LPL activity, and total VLDL hydrolysis. Considering this work is a pilot study, increasing the sample size may substantiate the results of the study. In contrast to previous studies, this work applied ITC for LPL activity measurement, thus avoiding the use of synthetic and radioactive substrates, and providing an alternative method to study LPL under in vivo-like conditions.

In summary, our results show that there was no significant difference in LPL activity measured in the fasted blood serum of vegans and omnivores. Interestingly, there were individuals in both groups that had similar TG levels but considerably different measured LPL activities and total VLDL TG hydrolysis. These results show that the vegan diet has no effect on blood sera as an environment for TRL hydrolysis by LPL; rather, there are other factors to consider besides dietary restrictions. Thus, following a vegan diet does not play a role in the context of vascular lipolysis in healthy individuals as compared to omnivores. Future research is warranted with individuals with similar TG levels but vastly different LPL activities through investigating such parameters as the physical properties of lipoproteins, HDL-C levels, and fatty acids in the composition of lipoprotein lipids.

## Figures and Tables

**Table 1:** Average exogenous LPL activity and blood parameters of both groups.

	LPL Activity ^1^ (µW/µg)	ApoA-I (g/L)	ApoB(g/L)	HDL-C (mmol/L)	TC ^2^ (mmol/L)	LDL-C ^3^ (mmol/L)	TG (mmol/L)
Vegan group N = 17	56.53 ± 17.97	1.61 ± 0.19	0.70 ± 0.22	1.53 ± 0.26	4.12 ± 0.82	2.33 ± 0.87	0.69 ± 0.35
Omnivore group N = 14	57.26 ± 18.03	1.61 ± 0.23	0.84 ± 0.21	1.52 ± 0.29	4.81 ± 0.85	3.02 ± 0.97	0.67 ± 0.31

^1^ Average exogenous LPL activity in blood sera of participants (heat released is proportional to fatty acids released per µg of protein). ^2^ *p* value < 0.05. ^3^ *p* value < 0.05.

**Table 2:** Participants with similar TG levels but different measured LPL activities.

Participant *	TG (mmol/L)	LPL Activity (µW/µg)	Difference *p* < 0.05
OMF231	0.33	22.57 ± 2.31	2.2-fold
OMF401	0.33	49.96 ± 3.72	
OMF222	0.39	36.10 ± 5.82	1.8-fold
VGNM211	0.39	65.83 ± 5.70	
OMF321	0.53	29.91 ± 3.85	2.8-fold
OMF351	0.53	84.77 ± 8.76	
OMF251	0.59	49.88 ± 6.70	1.7-fold
VGNF311	0.60	88.19 ± 8.37	
VGNF331	1.02	35.93 ± 0.39	2.5-fold
VGNM251	1.05	90.77 ± 5.17	

* Codes consist of diet (VGN—vegan; OM—omnivore), sex (M—male; F—female) and age.

## Data Availability

Data are contained within the article or Supplementary Material.

## Supplementary Materials

The following supporting information can be downloaded at: https://www.mdpi.com/article/10.3390/nu15122755/s1, Figure S1: Main dietary fat sources of both groups.

## Appendix A

Google colab code for chromatogram analysis.

The code written and used for chromatogram analysis can be found here:

https://colab.research.google.com/drive/1c8kN9M4xeQfpeNMJ0Fww6LxKXTREZzSZ?usp=sharing, accessed on 23 May 2023.
