# Supplementary material for: Lipoprotein Lipase Activity Does Not Differ in the Serum Environment of Vegans and Omnivores
Source: Nutrients. 2023 Jun 15;15(12):2755. doi: 10.3390/nu15122755 (PMC10303673; doi:10.3390/nu15122755)
Supplement: Supplementary file 1 [file nutrients-15-02755-s001.zip › nutrients-2439273-supplementary.pdf]

nutrients

MDPI

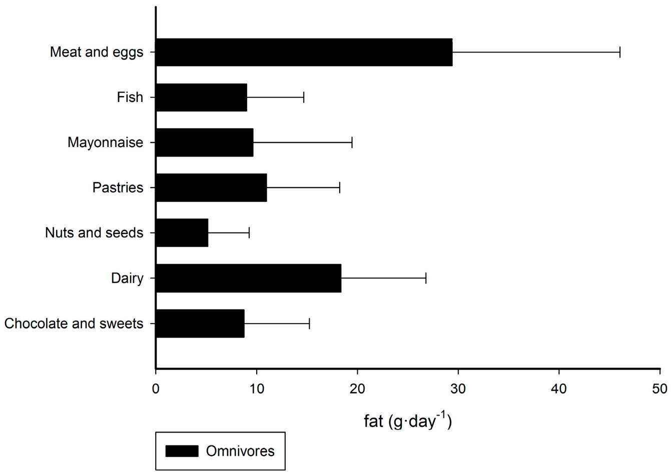

(A)

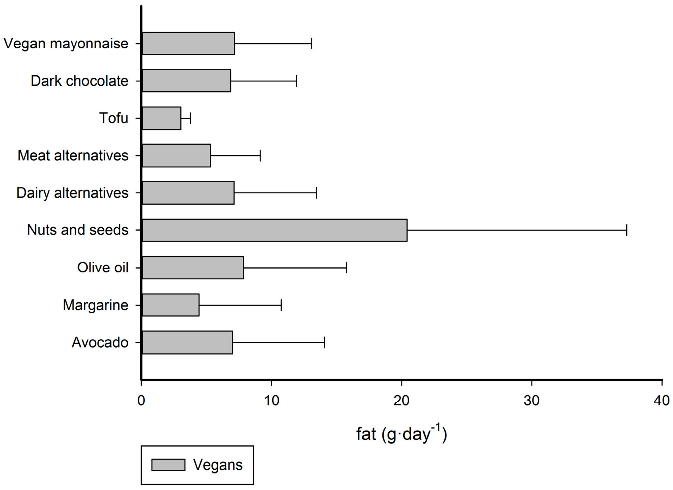

(B)

Figure S1. Mean daily consumption of dietary fat sources in both groups. The y-axis shows the main dietary fat sources consumed, while the x-axis shows the median daily amount of fat consumed from fat sources. (**A**) The main dietary fat sources in omnivore participants. Meat, eggs and dairy contributed the most to daily fat intake in omnivore participants; (**B**) The main dietary fat sources in vegan participants. Nuts and seeds were the main source of daily fat intake for vegans, followed by olive oil and dairy alternatives.
